# Editorial: Insights in microbial symbioses: 2022/2023

**DOI:** 10.3389/fmicb.2024.1367452

**Published:** 2024-01-23

**Authors:** Robert Czajkowski, Lifeng Zhu, Chih-Horng Kuo, Zhiyong Li

**Affiliations:** ^1^Laboratory of Biologically Active Compounds, Intercollegiate Faculty of Biotechnology UG and MUG, University of Gdansk, Gdansk, Poland; ^2^School of Medicine & Holistic Integrative Medicine, Nanjing University of Chinese Medicine, Nanjing, China; ^3^Evolutionary and Functional Genomics of Symbiotic Bacteria, Institute of Plant and Microbial Biology, Academia Sinica, Taipei, Taiwan; ^4^State Key Laboratory of Microbial Metabolism, School of Life Sciences and Biotechnology, Shanghai Jiao Tong University, Shanghai, China

**Keywords:** symbiosis, interaction, ecology, microbes, environment, holobiont

In recent years, significant improvements have been made in understanding microbial symbiosis, unraveling the complex connections between microorganisms and their hosts (Hays et al., 2015). Researchers explored symbiotic relationships in agroecosystems and insect-microbe interactions, revealing the diversity of microbial symbionts with potential applications in biofuel production and waste management (Aschenbrenner et al., 2016). Studies extended to extreme environments have uncovered novel symbiotic relationships in deep-sea hydrothermal vents and hypersaline basins, increasing our understanding of microbial adaptability and potential extraterrestrial life (Grzymski et al., 2008). Likewise, exploring gut microbiomes in various animals elucidates the delicate balance between hosts and resident microbes. These findings affect ecological conservation, sustainable agriculture, and personalized medicine (Sariola and Gilbert, 2020). As research continues, the intricate relationships between microorganisms promise to reveal more surprises and applications in the years ahead (Duperron, 2016).

This editorial explores the diverse range of topics covered in the seven articles under the Frontiers Research Topic: *Insights in microbial symbioses: 2022/2023*, each contributing to the ever-expanding areas of microbe-environment interactions.

Timmusk et al. article addresses the challenges imposed on land use and agriculture by global climate change. It underscores the pivotal role of microbial communities, particularly rhizobacteria, in shaping plant fitness, and agroecosystem biodiversity. The article supports a paradigm shift in our perspective, emphasizing that microbiomes define plant phenotypes, providing genetic variability crucial for the resilience of agroecosystems in the face of environmental changes.

The article by Schwarz et al. delves into the intricate symbiotic relationships between insects and their microbial companions, and it focuses on wood digestion in the passalid beetle *Odontotaenius disjunctus*. This study sheds light on the significance of microhabitats and reveals a diverse fiber-associated microbiome. The findings highlight insects' diverse evolutionary paths to adapt to wood-feeding, offering a deeper understanding of these complex ecological interactions.

The third article by Li et al. introduces an innovative approach to unraveling interspecies interactions at the genome-wide level. By integrating Lotka-Volterra equations into a systems mapping model, the study explores how the genes of coexisting species shape community structures and functions. Through a co-culture experiment involving *Escherichia coli* and *Staphylococcus aureus*, the researchers identify significant quantitative trait loci (QTL) combinations, providing a comprehensive view of the genetic mechanisms driving community dynamics and evolution.

The fourth article, authored by Zhang et al., focuses on the distribution patterns and traits of native rhizobia associated with *Pisum sativum* in Hebei Province, China. In a region experiencing an expansion of pea production, the study identifies distinct rhizobial communities and their efficiency in forming symbiotic partnerships with peas. This research offers valuable insights for optimizing crop breeding programs and enhancing the sustainability of legume cropping systems.

The fifth article, written by Yu et al., addresses the decline in yield and quality of *Gastrodia elata* Bl due to asexual reproduction. By isolating and identifying suitable germination fungi, particularly *Mycena purpureofusca*, the study provides a mechanism to enhance the yield of *G. elata* Bl. f. *glauca*. The research improves production performance and increases our understanding of the complex relationship between microbial communities and plant health.

The sixth article by Zhao et al. presents a comprehensive analysis of gut pathogens in different populations of giant pandas, both captive and wild. The study identifies unique pathogenic bacteria and virulence factors, unraveling their role in intestinal diseases that threaten the health and survival of these iconic animals. The findings contribute to our understanding of panda health and the development of effective conservation measures.

The last article gathered in this Research Topic was authored by Medina-Chávez et al.. This article explores microbial syntrophy in the extreme Cuatro Cienegas Basin. The study unveils a co-culture of a halophilic archaeon and a marine halophilic bacterium, emphasizing their shared characteristics and enhancing symbiotic association. Through genomic analysis, the research aims to uncover insights into the evolution of halophilic microorganisms and their remarkable adaptations to high-salinity environments.

In conclusion, these seven articles collectively offer a glimpse into the current state of the growing field of microbial symbiosis. Although the presented articles stand for a distinct field and cover various topics, each contribution adds a layer to our understanding of environmental microbial interactions. The decade ahead promises further revelations and breakthroughs in microbial symbiosis.

## Author contributions

RC: Conceptualization, Writing – original draft, Writing – review & editing. LZ: Writing – original draft, Writing – review & editing. C-HK: Writing – original draft, Writing – review & editing. ZL: Writing – original draft, Writing – review & editing.
